# Complete genome sequence of the saprophytic actinomycete *Rhodococcus (Prescottella) soli* DSD51W^T^, closely related to the multi-host pathogen *Rhodococcus (Prescottella) equi*

**DOI:** 10.1128/mra.00597-24

**Published:** 2024-10-29

**Authors:** Zeynep Yerlikaya, Raúl Miranda-CasoLuengo, Peadar Ó Gaora, Wim G. Meijer

**Affiliations:** 1 UCD School of Biomolecular and Biomedical Science and UCD Conway Institute, University College, Dublin, Ireland; 2 Department of Microbiology, School of Veterinary Medicine, Firat University, Elazığ, Türkiye; California State University, San Marcos, California, USA

**Keywords:** *Rhodococcus*, *Prescottella*, genome analysis

## Abstract

*Rhodococcus (Prescottella) soli* strain DSD51W^T^ is an aerobic, non-spore-forming, non-motile actinomycete isolated previously from soil collected from Kyoto Park, Japan, using a resuscitative technique. Here, we report the complete, circular genome sequence of *R. soli* DSD51W^T^. We employed a hybrid approach using Illumina and Oxford Nanopore platforms.

## ANNOUNCEMENT

*Rhodococcus (Prescottella) soli* species were isolated from pristine soil collected from Kyoto Park, Japan (1), and from glyphosate enrichment cultures that used herbicide-treated soil from Vietnam as inoculum (2). *R. soli*, *Rhodococcus (Prescottella) agglutinans*, *Rhodococcus (Prescottella) defluvii*, *Rhodococcus (Prescottella) subtropica,* and *Rhodococcus (Prescottella) equi* form a subclade in the *Rhodococcus* radiation (1, 3–6). Phylogenomic arguments are used for and against the placement of these species within the new genus *Prescottella* or conversely to maintain their placement within the genus *Rhodococcus* (3, 7–11). Although *Prescottella equi* and *Rhodococcus equi* are validly published names, the *R. equi* community voted to retain the genus *Rhodococcus* for this subclade (7). *R. soli* is closely related to *R. equi,* which is the only animal and human pathogen within the *Rhodococcus* radiation (1, 2, 10). The availability of the closely related *R. soli* genome, therefore, allows the identification of non-overlapping traits underpinning *R. equi* virulence as well as the evaluation of the phylogenomic position of the genus *Rhodococcus (Prescottella).*

We here announce the complete genome sequence of the *R. soli* type strain DSD51W^T^ (=KCTC 29259^T^=JCM 19627^T^=DSM 46662^T^=KACC 17838^T^), which was obtained from the Leibniz Institute DSMZ, Germany. *R. soli* was grown at 30°C for 48–72 h under aerobic conditions in tryptone soy broth (Oxoid) and tryptone soy agar (Oxoid). Approximately 5 × 10^9^ CFU were resuspended in 120 µL of Tris-EDTA buffer with lysozyme (0.1 mg/mL), Metapolyzyme (Sigma-Aldrich), and RNase A (0.1 mg/mL), incubated for 25 min at 37°C, followed by the addition of proteinase K (0.1 mg/mL) and SDS (0.5%), and incubated for 5 min at 65°C. Genomic DNA was purified using an equal volume of SPRI beads (Beckman). DNA sequencing was carried out on Illumina (NovaSeq 6000 using 2 × 250 bp) and Nanopore (GridION with R10.4.1 flowcells) platforms at MicrobesNG. Libraries for Illumina sequencing were prepared using the Nextera XT Library Prep Kit (Illumina) according to the manufacturer’s protocol. Long-read genomic DNA libraries for GridION sequencing were prepared with the Oxford Nanopore SQK-RB114.96 kit (ONT) using 200–400 ng of high-molecular-weight DNA resulting in 30,638 ONT reads. The average ONT read length was 11,453.47 bp. Illumina reads (*n* = 1,023,562) were adapter-trimmed using Trimmomatic version 0.30 with a sliding window quality Q15 cutoff (12). To achieve a complete and circular genome, long reads from ONT were integrated with Illumina reads using Unicycler v0.4.0 (13). This hybrid assembly allowed Unicycler to complete and circularize the genome by combining and refining the contigs. Overlapping ends were identified and trimmed by Unicycler, resulting in a single, complete circular chromosome. The final assembly was 5,475,811 bp in length with an average GC content of 68.65%. BUSCO v5.4.7 (14) provided a completeness score of 100%. Genome annotation was performed using the PGAP v6.7 resource of GenBank (15). The final assembly contains 4,936 protein-coding sequences, 71 pseudo-genes, 52 tRNAs, 15 rRNAs, and 3 ncRNA. Average nucleotide identity (ANI) was calculated using the fastANI tool v.1.33 (16). *R. soli* DSD51W^T^ has 85.37% ANI with *R. equi* 103S (GenBank FN563149.1).

## Data Availability

The genome sequence of *R. soli* DSD51W^T^ was deposited in GenBank under the accession number CP157276. The raw sequence reads were deposited in the SRA under the accession numbers SRR29248331 (GridION) and SRR29248330 (Illumina NovaSeq 6000). The associated BioProject and BioSample accession numbers are PRJNA1116094 and SAMN41522625, respectively

## AFTER EPUB

*[This article was published on 29 October 2024 with an amendment to the genus name. The genus name was corrected throughout in the current version, posted on 12 November 2024.]*
